# Association between measures of adiposity and blood pressure levels in adult Cameroonians

**DOI:** 10.1002/hsr2.259

**Published:** 2021-05-03

**Authors:** Anastase Dzudie, Nelson Njedock, Jerome Boombhi, Hamadou Ba, Sylvie Ndongo Amougou, Felicite Kamdem, Blaise Barche, Archange Nzali, Armel Njomou, Calypse Ngwasiri, Meh Martin Geh, Marcel Azabji, Alain Chichom Mefire, Alfred K. Njamnshi, Samuel Kingue, Jean Philippe Empana, Eugene Sobngwi, Laurent Serges Etoundi Ngoa, Andre Pascal Kengne

**Affiliations:** ^1^ Faculty of Medicine and Biomedical Sciences University of Yaounde 1 Yaounde Cameroon; ^2^ Clinical Research Education Networking and Consultancy (CRENC) Douala Douala Cameroon; ^3^ Service of Internal Medicine and Subspecialties, Douala General Hospital Douala Cameroon; ^4^ Service of Internal Medicine, Hopital Laquintinie Douala Cameroon; ^5^ St Mary Soledad Catholic Hospital Bamenda Cameroon; ^6^ Faculty of Health Sciences University of Buea Yaounde Cameroon; ^7^ Brain Research Africa Initiative (BRAIN) Geneva Switzerland/Yaoundé Cameroon; ^8^ Paris Cardiovascular Research Center (PARCC) Université de Paris, INSERM, Integrative Epidemiology of Cardiovascular Diseases Team Paris France; ^9^ Non‐Communicable Diseases Research Unit Medical Research Council Cape Town South Africa

**Keywords:** adiposity, blood pressure, Cameroon, hypertension, ROC curve

## Abstract

**Introduction:**

Several anthropometric measurements are variably recommended to assess adiposity in routine practice, with less agreement on their comparative performance. We assessed and compared the relationship of seven anthropometric measures of adiposity—waist circumference (WC), waist‐to‐height ratio (WHtR), Body Mass Index (BMI), Ponderal Index (PI), Conicity Index (C index), A Body Shape Index (ABSI), and Body Roundness Index (BRI)—with blood pressure (BP) levels and prevalent hypertension in adult Cameroonians.

**Methods:**

Data were collected as Cameroon's contribution to the global May Measurement Month 2017(MMM17) survey. Participants were nonpregnant adults, who had no BP measurement in the past year and with no prior hypertension diagnosis. Hypertension was defined as systolic BP ≥140 mm Hg and/or diastolic ≥90 mm Hg. Odds ratios (ORs) for the presence of hypertension per 1 SD increase in each adiposity metrics were estimated in separate logistic regression models. Assessment and comparison of discrimination used the area under the receiver operating characteristics curve (AUC) and nonparametric methods.

**Results:**

We included 14 424 participants (8210 [58.25%] female; 39.84 ± 14.33 years). There was a graded association between measures of adiposity and prevalent screen‐detected (newly diagnosed) hypertension, with effect sizes being mostly within the same range across measures of adiposity. AUC for hypertension prediction ranged from 0.709 with PI to 0.721 with BRI for single measures, and from 0.736 to 0.739 with combinations of measures of adiposity.

**Conclusion:**

WC, WHtR, and BRI were strongly associated with BP and better predicted prevalent hypertension, with effects enhanced with the inclusion of BMI.

**Abbreviations**
ABSI: A body Shape Index
BMI: Body Mass Index
BRI: Body Roundness Index
C Index: Conicity Index
PI: Ponderal Index
WC: Waist circumference
WHtR: waist‐to‐height ratio

## 1 INTRODUCTION

Hypertension is the most common modifiable risk factor for cardiovascular disease (CVD).^1^ The World Health Organization (WHO) estimated that Africa has the highest prevalence of hypertension at about 40% in adults aged 25 years and older in some countries, compared to 35% to 40% elsewhere around the world.^1^, ^2^

In its 10‐point action plan to reduce the burden of high blood pressure (BP) on the continent, the Pan African Society of Cardiology (PASCAR) called on urgent investment in population‐level interventions for preventing hypertension occurrence, such as reducing salt intake and obesity.^3^

Globally, excess weight is associated with the development of hypertension.^4^, ^5^ In 2010, 27% of adult Africans were overweight, and 8% were obese.^6^ Body Mass Index (BMI) is the most widely used marker to diagnose obesity, and has often been overlooked as a proxy of total adiposity. Central adiposity, an accumulation of body fat in the lower torso around the abdominal area, has been associated with an increased risk of heart disease, dementia, type 2 diabetes, and hypertension. Increasing evidence supports the superiority of measures of central adiposity especially waist‐to‐height ratio (WHtR), over BMI, in discriminating cardiovascular risk in both men and women^7^ and combining BMI with other indices has also been shown to improve the prediction of cardiovascular risk.^8^ Recently, new indices of central obesity have been proposed and are being explored on their abilities to predict cardiovascular risk and all‐cause mortality.^9^, ^10^ However, findings have been controversial and studies comparing the discriminatory capacity of different indices of obesity on health status and outcomes in African populations are lacking.

We aimed to assess the relationship of seven anthropometric measures of adiposity—waist circumference (WC), WHtR, BMI, Ponderal Index (PI), Conicity Index (C index), A Body Shape Index (ABSI), and Body Roundness Index (BRI) with BP levels and prevalent hypertension in adult Cameroonians.

## 2 MATERIALS/SUBJECTS AND METHODS

The current study is based on secondary analysis of data for Cameroonians who took part in the May Measure Month 2017 (MMM17). Specific methods for the MMM study, have been described in detail elsewhere.^11^, ^12^ Briefly, participants were self‐selected adult men and women. Sampling was consecutive and exhaustive. The study protocol conformed to the ethical guidelines of the 1975 Declaration of Helsinki: Ethical clearance was obtained from the Cameroon national ethics committee; various administrative authorizations were obtained from registered sites and informed consent was obtained from each participant. The following data were collected: date of last BP measurement; date of birth; gender; history of antihypertensive medication, diabetes (yes/no), smoking (yes/no), heart attack, stroke, and alcohol consumption; height, weight, WC, and three BP and heart rate measurements.

Participants aged less than 18 years, self‐reported pregnant women and those on antihypertensive medications were excluded from the current analysis.

### 2.1 Measures of adiposity

Weight to the nearest kilogram was measured using calibrated weighing scales; while height to the nearest centimeter was measured using a stadiometer. Then, BMI (kg/m^2^) was calculated as weight (kg)/height^2^ (m^2^). WC was measured, to the nearest centimeter, in the horizontal plane midway between lowest ribs and the superior borders of the iliac crests using measuring tapes. WHtR was estimated as WC (m) divided by the height (m), and PI as weight (kg) divided by Height*height*height(m^3^).^13^ Other calculations done are described below:

1. The C index, an index of adiposity which derives from WC, weight, and height and is calculated using the formula:

$C\;\text{index}=\frac{\mathrm{WC}\;\left(m\right)}{0.109\times \sqrt{\frac{\mathrm{Wt}\;\left(\mathrm{kg}\right)}{\mathrm{Ht}\;\left(m\right)}}}$ ^14^

2. ABSI is an index of adiposity, which combines information from WC, height, and weight and was calculated as:

$\text{ABSI}=\frac{\mathrm{WC}\;\left(m\right)}{{\mathrm{BMI}}^{2/3}\left(\mathrm{kg}/m^{2}\right)\;\;{\mathrm{Ht}}^{1/2}\left(m\right)}$ ^15^

3. The BRI is an index of adiposity calculated using the formula below with the use of height, weight, WC, and hip circumference.

$\mathrm{BRI}=364.2-365.5\times \sqrt{1-\left(\frac{{\left(\mathrm{WC}\left(m\right)/\left(2\pi \right)\right)}^{2}}{{\left(0.5\mathrm{Ht}\left(m\right)\right)}^{2}}\right)}$ ^16^

To streamline all the above calculations, we used a web‐based calculator.

### 2.2 Blood pressure profile assessment

Most BP were measured with automated electronic Omron sphygmomanometer, by trained health care providers following BP measurement guidelines. Prior to BP measurement, participants were seated for at least 5 minutes. Three BP readings were taken and recorded with a minimum of 1 minute between readings. Hypertension was defined as systolic and/or (diastolic) BP ≥140 mm Hg (≥90 mm Hg).^17^

### 2.3 Statistical analysis

Statistical analysis was done using “R” v.3.4.2. Only the first of the three BP readings were used for the main analysis presented in this report, due to the large number of missing data for the second and third BP readings. A sensitivity analysis was conducted using the average of the second and third BP readings in participants with available data on the three readings. The association between anthropometric indices was examined using the pairwise Pearson's correlation and interpreted based on the classification of correlation coefficients.^18^ The Pearson's correlation between BP and measures of adiposity were compared with Steiger's test. Separate logistic regressions models were used to assess the independent association between screen‐detected hypertension (defined as hypertension diagnosed during the screening in a participant not previously known to have hypertension) and each anthropometric index. The odds ratio (OR) and the 95% confidence interval (CI) were given per SD increase in anthropometric measure, but also across quintiles of adiposity measures using the lowest quintile as reference. All models were adjusted for age and gender. The ability of anthropometric indices in isolation or in combination to discriminate between participants who had and those who did not have screen‐detected hypertension (SDH) was assessed and compared using the area under the receiver operating characteristic curves (AUC), and nonparametric methods.

## 3 RESULTS

### 3.1 Data available

**FIGURE 1:**
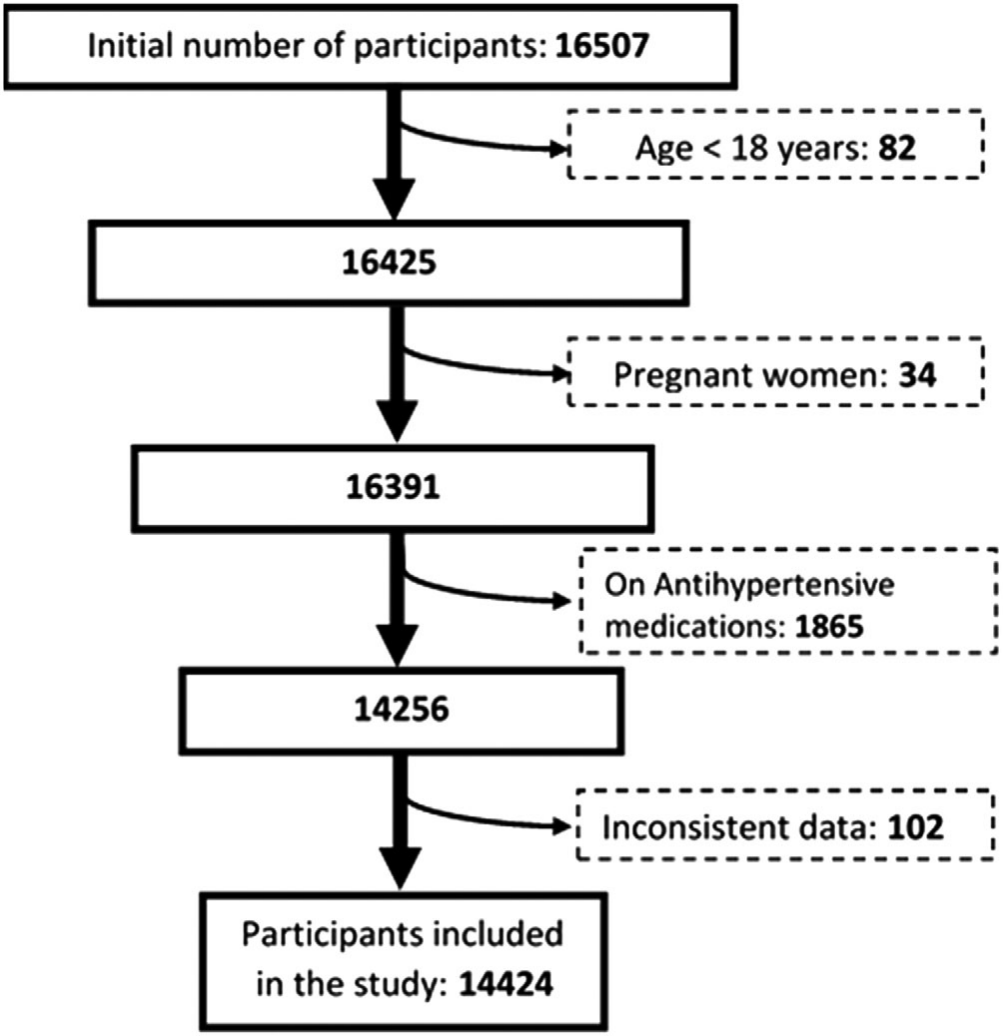
Flow chart demonstrating how exclusion criteria were applied

Overall, 16 507 participants from nine regions of Cameroon responded to the invitation for screening, 2083 were excluded for reasons described in Figure 1, and the remaining 14 424 were included in the main analysis.

### 3.2 General Characteristics of the study participants

The general characteristics of the 14 424 included participants (41.7% men) are summarized in Table 1. The mean age of study participants was 39.8 years overall, 40.1 years for men, and 39.7 years for women (*P* = .074 for gender difference). The mean WC, BMI, WHtR, SBP, and DBP were 89.4 (±14.1) cm, 27.9 (±9.2) kg/m^2^, 0.54 (±0.09), 124. 7 (±20.5) mm Hg, and 78.2 (±13.2) mm Hg, respectively. Compared to women, men were more likely to smoke (2.6% vs 8.6%, *P* < .001), and to regularly consume alcohol (44.0% vs 53.6%, *P* < .001). The proportion of individuals with SDH was 29.3% in men and 22.0% in women, respectively.

**TABLE 1:** Distribution of the baseline characteristics of the study population according to gender

Variables	Females	Males	*P*‐value	Total
N (%)	8210 (58.25)	5885 (41.75)		14 424
Age (years)	39.7 (±14.5)	40.1 (±14.1)	.074	39.8 (±14.3)
	37.0 (28.0‐49.0)	38.0 (29.0‐49.0)	.009	37.0 (28.0‐49.0)
Weight (kg)	74.2 (±15.7)	76.3 (±14.4)	<.0001	75.1 (±15.2)
	73 (63‐83)	75 (66‐85)	<.0001	74.0 (64‐84)
Height (cm)	162.5 (±11.9)	170.5 (±12.0)	<.0001	165.9 (±12.6)
	163 (159‐168)	172 (167‐176)	<.0001	167 (160‐172)
Waist circumference (cm)	89.6 (±14.6)	89.2 (±13.1)	.205	89.4 (±14.1)
	89 (80‐99)	88 (80‐98)	.061	890 (80–98)
BMI (kg/m^2^)	28.7 (±9.5)	26.8 (±8.8)	<.0001	27.9 (±9.2)
	27.3 (23.7‐31.2)	25.5 (22.9‐28.8)	<.0001	26.5 (23.3‐30.1)
Waist‐height‐ratio	0.55 (±0.09)	0.52 (±0.08)	<.0001	0.54 (±0.09)
	0.54 (0.45‐0.60)	0.52 (0.47‐0.57)	<.0001	0.53 (0.48‐0.59)
Ponderal Index (kg/m^3^)	18.4 (±12.7)	16.3 (±11.0)	<.0001	17.5 (±15.9)
	16.7 (14.5‐19.3)	14.8 (13.3‐17.0)	<.0001	15.9 (13.8‐18.3)
Conicity Index (m^2/3^/kg^1/2^)	1.21 (±0.15)	1.22 (±0.13)	.092	1.21 (±0.14)
	1.21 (1.14‐1.29)	1.21 (1.14‐1.29)	.455	1.21 (1.14‐1.29)
A body Shape Index (m^7/6^/kg^2/3^)	0.095 (0.011)	0.096 (±0.011)	.0005	0.095 (±0.011)
	0.095 (0.089‐0.100)	0.095 (0.090‐0.101)	.002	0.095 (0.090–0.101)
Body Roundness Index	4.49 (±2.05)	3.94 (±1.81)	<.0001	4.28 (±1.97)
	4.25 (3.14‐5.51)	3.69 (2.77‐4.80)	<.0001	4.00 (2.96‐5.20)
Systolic blood pressure (mm Hg)	121.5 (±20.4)	128.1 (±19.9)	<.0001	124.7 (±20.5)
	118 (108‐131)	126 (116‐139)	<.0001	122 (110‐135)
Diastolic blood pressure (mm Hg)	77.5 (±13.1)	79.4 (±13.4)	<.0001	78.2 (±13.2)
	76 (69‐85)	78 (71‐87)	<.0001	77 (70‐86)
Heart rate (bpm)	81 (±14)	77 (±13)	<.0001	79 (±14)
	80 (72‐89)	76 (68‐85)	<.0001	78 (70‐88)
History of diabetes, n (%)	n = 8163	n = 5820	.143	n = 14 305
Yes	178 (2.2)	149 (2.6)		341 (2.4)
BMI, n (%)	n = 4963	n = 6647	<.001	n = 11 834
<18.5 kg/m^2^	125 (1.88)	110 (2.22)		241 (2.04)
18.5‐25 kg/m^2^	2101 (31.61)	2132 (42.96)		4330 (36.59)
25‐30 kg/m^2^	2295(34.53)	1805, (36.37)		4163 (35.18)
30‐35 kg/m^2^	1291(19.42)	670 (13.50)		2000 (16.90)
35‐40 kg/m^2^	543 (8.17)	159 (3.20)		712 (6.01)
>40 kg/m^2^	292 (4.39)	87 (1.75)		388(3.28)
Smoking n (%)	n = 8172	n = 5856	<.001	n = 14 349
Yes	211 (2.6)	509 (8.7)		740 (5.2)
Residence, n (%)	n = 8158	n = 5847	<.001	n = 14 361
Urban	6657 (81.6)	5078 (86.4)		12 021 (83.7)
Alcohol consumption, n (%)	n = 8168	n = 5824	<.001	n = 14 311
Never or rarely	4329 (53.0)	2533 (43.5)		7023(49.1)
Less than once a week	244 (3.0)	167 (2.9)		413(2.9)
Regularly	3595 (44.0)	3124 (53.6)		6875(48.0)

*Note*: Values are count (percentages, %) and mean (±SD, SD); or median and 25th to 75th percentiles. NA, not applicable; proportions are computed across columns. Means are compared using independent samples *t* test and medians using the Wilcoxon‐Mann‐Whitney rank sum test, the rest of the characteristics are frequencies (percentage) compared using chi squared test or Fisher's exact test.

### 3.3 Relationship between anthropometric indices

The Pearson's correlation coefficients between anthropometric variables are summarized in Table 2. In general, women had slightly higher estimates of correlation coefficients between anthropometric indices than men. Correlation of BMI with C Index was nonsignificant in men (*r* = .02, *P* > .10), while correlations of BMI with ABSI and C Index, and that of C Index with PI were consistently negative in men and women, and always of lower magnitude, ranging from −0.18 to −0.05 (Table 2). All other correlation coefficients were positive ranging from 0.02 (BMI vs C Index) to 0.99 (ABSI vs C Index) in men, and from 0.07 (BMI vs C Index) to 0.99 (ABSI vs C Index) in women.

**TABLE 2:** Pearson's Correlation coefficients between anthropometric variables (superior lateral half, males; inferior lateral half, females)

	WC	WHtR	BMI	PI	C Index	ABSI	BRI
WC	1	0.85	0.51	0.28	0.79	0.69	0.83
WHtR	0.93	1	0.69	0.58	0.62	0.50	0.98
BMI	0.62	0.71	1	0.95	0.02*	−0.13	0.72
PI	0.32	0.56	0.94	1	−0.07	−0.18	0.63
C Index	0.78	0.69	0.07	−0.05	1	0.99	0.63
ABSI	0.66	0.56	−0.10	−0.16	0.99	1	0.51
BRI	0.90	0.98	0.73	0.63	0.65	0.53	1

Abbreviations: ABSI, A body Shape Index; BMI, Body Mass Index; BRI, Body Roundness Index; PI, Ponderal Index; C Index, Conicity Index; WC, waist circumference; WHtR, waist‐to‐height ratio.

^*^ *P* > .1; all the rest are significantly statistically different from zero *P* < .05.

### 3.4 Correlation between anthropometric indices and blood pressure levels

Unadjusted correlation coefficients between anthropometric measures and BP levels are shown in Table 3, separately for men and women. For both SBP and DBP and consistently in men and women, the higher correlations coefficients were always with WC, followed by BRI and WHtR. Correlation coefficients were generally of modest sizes, always positive within the same range in men and women, and ranged from 0.05 (PI vs DBP) to 0.26 (WC vs DBP) in men, and from 0.05 (ABSI vs DBP) to 0.23 (WC vs DBP) in women for DBP; and from 0.04 (PI vs SBP) to 0.26 (WC vs SBP) in men, and from 0.05 (PI vs SBP) to 0.24 (WC vs SBP) in women for SBP (Table 3). In both men and women, the Steiger test showed that correlations of SBP with WC were always significantly higher than with other anthropometric measures (all *P* < .0001) with the exception of WHtR (both *P* ≥ .116) and BRI (both *P* ≥ .052). The pattern was similar for DBP in women, while in WC vs DBP was significantly higher than BRI vs DBP (*P* = .019), and borderline higher than WHtR vs DBP (*P* = .050). Other correlation coefficients comparisons are shown in Table 3.

**TABLE 3:** Pearson's Correlation coefficients between anthropometric and systolic and diastolic blood pressure (superior lateral half, males; inferior lateral half, females) and *P*‐value of the comparison between them

	r(Males)	WC	WHtR	BMI	PI	Cndex	ABSI	BRI
r(Females)	DBP	0.26	0.21	0.11	0.05	0.14	0.10	0.20
WC	0.23	**1**	0.050	<0.0001	<0.0001	<0.0001	<0.0001	0.019
WHtR	0.21	0.327	**1**	<0.0001	<0.0001	0.0099	0.0001	0.705
BMI	0.12	<0.0001	<0.0001	**1**	0.002	0.212	0.679	0.0001
PI	0.06	<0.0001	<0.0001	0.0004	**1**	0.0002	0.040	<0.0001
Cindex	0.10	<0.0001	<0.0001	0.3137	0.0451	**1**	0.153	0.027
ABSI	0.05	<0.0001	<0.0001	0.0004	0.618	0.027	**1**	0.0003
BRI	0.21	0.327	1	<0.0001	<0.0001	<0.0001	<0.0001	**1**
r(Males)	SBP	0.26	0.22	0.11	0.04	0.13	0.09	0.21
WC	0.24	**1**	0.116	<0.0001	<0.0001	<0.0001	<0.0001	0.052
WHtR	0.22	0.325	**1**	<0.0001	<0.0001	0.0009	<0.0001	0.704
BMI	0.12	<0.0001	<0.0001	**1**	0.0003	0.406	0.408	<0.0001
PI	0.05	<0.0001	<0.0001	0.0000	**1**	0.0002	0.040	<0.0001
Cindex	0.12	<0.0001	<0.0001	1	0.0004	**1**	0.154	0.003
ABSI	0.08	<0.0001	<0.0001	0.044	0.134	0.075	**1**	<0.0001
BRI	0.23	0.622	0.631	<0.0001	<0.0001	<0.0001	<0.0001	**1**

*Note*: Bold writing was used to highlight the item line, which can also be made simple

Abbreviations: ABSI, A body Shape Index; BMI, Body Mass Index; BRI, Body Roundness Index; DBP, diastolic blood pressure; PI, Ponderal Index; C Index, Conicity Index; *r*, correlation coefficient; SBP, systolic blood pressure; WC, waist circumference; WHtR, waist‐to‐height ratio.

### 3.5 Association between anthropometric indices and screen‐detected hypertension

There was a gradual increase in the odds of prevalent hypertension across increasing quintiles of anthropometric variables, with the pattern being however less apparent for ABSI, and C index (Table 4). With the exception of these two variables, the adjusted odd ratio comparing the top with the lowest quintiles was within the same range across anthropometric variables, with point estimates ranging from 1.16 to 1.18 and the confidence intervals around these estimates always overlapping. A SD higher level of anthropometric variables was associated with adjusted odds ratio ranging from 1.01 (95% CI 0.95‐1.07) for ABSI, to 1.39 (1.31‐1.48) for WC. WHtR [1.37 (1.29‐1.46)] and BRI [1.34 (1.26‐1.42)] had the second and the third highest OR associated with a SD change in their levels, in relation with prevalent hypertension (Table 4).

**TABLE 4:** Odds ratio and 95% confidence intervals for the presence of hypertension across quintiles of anthropometric variables and for a SD increase in the anthropometric variables

Variable		Quintile 1	Quintile 2	Quintile 3	Quintile 4	Quintile 5	OR (95%CI) per SD change	AUC (95%CI)
Waist circumference	Median (cm)	74	82	89	96	107		
	OR (95% CI)	Reference	0.99 (0.96‐1.02)	1.02 (0.99‐1.05)	1.11 (1.07‐1.14)	1.16 (1.13‐1.20)	1.39 (1.31‐1.48)***	0.716 (0.703‐0.730)
Waist to height ratio	Median	0.44	0.49	0.53	0.58	0.65		
	OR (95% CI)	Reference	0.99 (0.96‐1.02)	1.06 (1.03‐1.10)	1.08 (1.05‐1.12)	1.18 (1.14‐1.22)	1.37 (1.29‐1.46)***	0.720 (0.706‐0.734)
Body mass index	Median (kg/m^2^)	21.0	23.9	26.5	29.3	34.5		
	OR (95% CI)	Reference	1.02 (0.99‐1.04)	1.06 (1.03‐1.08)	1.11 (1.09‐1.14)	1.16 (1.14‐1.19)	1.20 (1.15‐1.25)***	0.714 (0.704‐0.725)
Ponderal index	Median	12.31	14.22	15.86	17.70	21.28		
	OR (95% CI)	Reference	1.01 (0.99–1.04)	1.07 (1.04‐1.10)	1.10 (1.07‐1.13)	1.16 (1.13‐1.19)	1.08 (1.03‐1.12)***	0.709 (0.698‐0.720)
Conicity index	Median	1.07	1.16	1.21	1.27	1.37		
	OR (95% CI)	Reference	1.00 (0.97‐1.03)	1.05 (1.02‐1.09)	1.05 (1.02‐1.09)	1.08 (1.04‐1.12)	1.09 (1.02‐1.16)*	0.717 (0.703‐0.731)
A body shape index	Median	0.084	0.091	0.095	0.099	0.106		
	OR (95% CI)	Reference	0.98 (0.95‐1.01)	1.03 (1.00‐1.06)	1.04 (1.00‐1.07)	1.03 (0.99‐1.06)	1.01 (0.95‐1.07)	0.716 (0.702‐0.731)
Body roundness index	Median	2.299	3.155	3.997	4.911	6.537		
	OR (95% CI)	Reference	0.99 (0.96–1.02)	1.06 (1.03‐1.10)	1.08 (1.05‐1.12)	1.18 (1.14‐1.22)	1.34 (1.26‐1.42)***	0.721 (0.707‐0.734)

Abbreviations: AUC, area under the receiver operating characteristic curves; OR, odds ratio (adjusted for age, gender, smoking, alcohol, diabetes mellitus). SD, standard deviation.

^*^ *P* < .05;

^***^ *P* < .001.

### 3.6 Discrimination of screen‐detected hypertension by adiposity indices

The discrimination capacities of single or combined anthropometric variables screen‐detected hypertension are shown in Table 4 and Table S1. The AUC of single adiposity variable ranged from 0.709 (0.698‐0.720) with PI to 0.721 (95%CI: 0.707‐0.734) with BRI, but AUC did not differ between the models. Models with two anthropometric variables combination had better discrimination capability than any model containing a single anthropometric variable, with AUC ranging from 0.736 to 0.739.

### 3.7 Sensitivity analysis

When the above analyses were repeated in the subset of participants with data available on more than one BP measurements, the pattern of results obtained using average BP across those measurements was mostly similar to those based on single BP measurement (Tables [Link], [Link], [Link]).

## 4 DISCUSSION

Studies assessing the performance of measures of adiposity as predictors of CVD risk in sub‐Saharan Africa are limited and most of those available have focused on BMI as predictors of hypertension. In this study, we compared measures of adiposity for their association with BP in a large population of self‐selected Cameroonian adults free of any history of diagnosed hypertension. WC, WHtR, and BRI emerged as the best predictor of screen‐detected hypertension, with indications that combining them with BMI further enhanced screen‐detected hypertension prediction, although the clinical importance of such improvement could be questioned. Because WC is much easy to acquire than WHtR and BRI, our findings suggest a focus on WC in routine setting is likely to capture the essential discriminatory power of adiposity in relation with hypertension, and likely CVD risk.

Measures of adiposity have shown a significant association with hypertension and other CVD risk factors in African populations.^19^, ^20^, ^21^ In accordance with these existing studies, our analyses showed that there was an association between all tested indices of adiposity and screen‐detected hypertension. These associations were mostly continuous, suggesting that clinical approach of using thresholds of those indices for CVD risk screening, do not optimally capture the discriminatory information from those markers. WC, WHtR, and BRI demonstrated a relatively stronger association with SDH when compared to other indices. These results are in support of the growing evidence that measures of visceral (or central) fat accumulation are better predictors of cardiovascular risks in Africans and Asians.^19^, ^20^, ^21^

A number of mechanisms to explain the stronger association of central fat accumulation with CVD risk have been postulated. One of the suggested mechanisms is that visceral adipose tissue has a higher degree of metabolic activity. Compared with subcutaneous peripheral adipose tissue, visceral adiposity has greater sympathetic innervation with a large number of β3‐adrenergic receptors. This facilitates a higher metabolic activity, and correlates well with other markers of cardio‐metabolic derangements.^22^, ^23^ Visceral fat has a stronger correlation with adipokines like leptins that have been implicated in the inflammatory pathophysiological hypothesis of CVD.^24^, ^25^

The relative performance of predictive models containing measures of adiposity was assessed by comparing AUCs. All the predictive models with single adiposity measures had good performance in predicting SDH. Though BRI and WHtR had highest AUCs, they were not significantly different from WC and BMI. Adiposity indices incorporating a measure of central obesity had better predictive performance than BMI and PI. Asian countries have explored the less frequently used measures of adiposity and have shown the superiority of indices of central obesity over those of overall obesity.^26^, ^27^ Nonetheless, the report from Cheung and collaborators was contradictory.^28^ In line with our findings, ABSI has been reported in many studies to poorly predict hypertension and other CVD risk factors.^26^, ^27^ There was no statistically significant difference between the ability of WHtR, BRI, and WC to predict hypertension and that of BMI. When models with combined adiposity measures were evaluated, BMI appeared to significantly improve the performance of all the other models with single adiposity measures. The combination of WC + C index appeared to be the best model in predicting SDH, with a significant difference when compared with all the other models. These findings are in contrast with previous reports. In Nigeria, Ononamadu and collaborators did not find any significant improvement in model prediction when WC or WHtR were added to BMI in adjusted models. In Cameroon, another study evaluating the combination of adiposity measures in predicting another cardiovascular risk factor, demonstrated that combining BMI to other indices did not improve prediction.^19^

We can thus speculate that though abdominal obesity is central to the presence of cardiovascular risk factors, the contribution of overall adiposity cannot be underestimated. The complex interaction between genetic predisposition, phenotypic characteristics, sociocultural, and environmental factors coupled with the differences in methodological approaches may also be suggested as the potential contributors to the observed differences across studies. The results of combining WC and C index in models has not been reported in previous studies and therefore deserve further exploration.

Our study has some limitations. This was a self‐selected population, which may not be a representative sample of the Cameroonian nation and hence true prevalence cannot be reported. BP screening took place on a single occasion, with the risk of false‐positive diagnoses of high BP. This is a cross‐sectional study and causal inference between adiposity marker and BP levels cannot be made. It remains however that our very large sample size has provided a very good statistical power to generate this very first detailed report on the association of measures of adiposity with BP levels and screen‐detected hypertension in an African population.

## 5 CONCLUSION

WC, WHtR, and BRI were strongly associated with BP and better predicted high BP risk. Given that these indices of central obesity (WC, WHtR, and BRI) are simple to measure and provides additional important information on metabolic risk, we recommend their systematic use in combination with BMI but not BMI alone to identify and monitor patients with high BP risks. Further research would identify the effectiveness of interventions on these indices to reduce chances of developing high BP in our population.

## CONFLICT OF INTEREST

The authors declare there is no conflict of interest.

## AUTHOR CONTRIBUTIONS

Study concept and design: Dzudie, Njedock, and Kengne

Acquisition of data: Njedock, Ba, Boombhi, Ndongo Amougou, Kamdem, and Dzudie

Analysis and interpretation of data: Dzudie, Njedock, Ba, Sobngwi, and Kengne

Drafting of the manuscript: Dzudie, Njedock, Etoundi Ngoa, and Kengne

Critical revision of the manuscript for important intellectual content: Kengne, Etoundi Ngoa, Empana, and Dzudie

Statistical analysis: Njedock, Dzudie, and Kengne

     All authors have read and approved the final version of the manuscript.

     Anastase Dzudie had full access to all of the data in this study and takes complete responsibility for the integrity of the data and the accuracy of the data analysis.

## TRANSPARENCY STATEMENT

Anastase Dzudie affirms that this manuscript is an honest, accurate, and transparent account of the study being reported; that no important aspects of the study have been omitted; and that any discrepancies from the study as planned (and, if relevant, registered) have been explained.

## Supporting information


**Table S1** Area under the receiver operating characteristic curve (AUC) and the percentage change in AUC with single and two by two combination of adiposity measures for their abilities to predict hypertension.

**Table S2** Odds ratios for a SD change measures and across quintiles of adiposity measures for predicting hypertension using average blood pressure adjusted for age and gender

**Table S3** Area under the receiver operating characteristics curve for single and combined adiposity measures using average blood pressure

**Table S4** correlation coefficients (r) between Systolic and diastolic blood (SBP and DBP) for male(M) and females (F)

## Data Availability

The data that support the findings of this study are available on request from the corresponding author. The data are not publicly available due to privacy or ethical restrictions.
