# Supplementary material for: Association between measures of adiposity and blood pressure levels in adult Cameroonians
Source: Health Sci Rep. 2021 May 3;4(2):e259. doi: 10.1002/hsr2.259 (PMC8093855; doi:10.1002/hsr2.259)
Supplement: Supplementary file 1 — Table S1 Area under the receiver operating characteristic curve (AUC) and the percentage change in AUC with single and two by two combination of adiposity measures for their abilities to predict hypertension. [file HSR2-4-e259-s001.docx]

| **Supplementary Table 1: Area under the receiver operating characteristic curve (AUC) and the percentage change in AUC with single and two by two combination of adiposity measures for their abilities to predict hypertension.** | | | | | | | | | | | | | | |
| --- | --- | --- | --- | --- | --- | --- | --- | --- | --- | --- | --- | --- | --- | --- |
|  |  |  |  |  |  |  |  |  |  |  |  |  |  |  |
|  |  |  |  |  |  |  |  |  |  |  |  |  |  |  |
|  | **AUC** | **WC** | **WHtR** | **BRI** | **BMI** | **PI** | **Cindex** | **ABSI** | **WC+BMI** | **WHtR+BMI** | **BRI+BMI** | **Cindex+BMI** | **ABSI+BMI** | **WC+Cindex** |
| **WC** | 0.716 (0.703 0.730) | NA | 0.58 | 0.63 | -0.26 | -0.98* | 0.12* | 0.037* | 2.87 | 2.82* | 2.837* | 2.84* | 2.84* | 3.191* |
| **WHtR** | 0.720 (0.706 -0.734) |  | NA | 0.62 | -0.83 | -1.55* | -0.46* | -0.54* | 2.27* | 2.23* | 2.24* | 2.24* | 2.24* | 2.59* |
|  |  |  |  |  |  |  |  |  |  |  |  |  |  |  |
| **BRI** | 0.721 (0.707 -0.734) |  |  | NA | -0.88 | -1.59* | -0.50* | -0.58* | 2.23* | 2.18* | 2.20* | 2.20* | 2.20* | 2.55* |
| **BMI** | **0.714(0.704 -0.725)** |  |  |  | NA | -0.72* | 0.38* | 0.30* | 3.13* | 3.09* | 3.10* | 3.10* | 3.11* | 3.45* |
| **PI** | **0.709 (0.698 -0.720)** |  |  |  |  | NA | 1.11 | 1.02* | 3.88* | 3.84* | 3.85* | 3.85* | 3.86* | 4.21* |
| **Cindex** | 0.717 (0.703 -0.731) |  |  |  |  |  | NA | -0.09 | 2.73* | 2.70* | 2.71* | 2.71* | 2.72* | 3.06* |
| **ABSI** | 0.716 (0.702 -0.731) |  |  |  |  |  |  | NA | 2.83* | 2.79* | 2.80* | 2.80* | 2.80* | 3.15* |
| **WC+BMI** | 0.737 (0.723 -0.750) |  |  |  |  |  |  |  | NA | -0.04 | -0.03 | -0.03 | -0.02 | 0.32* |
| **WHtR+BMI** | **0.736 (0.722 -0.750)** |  |  |  |  |  |  |  |  | NA | 0.01 | 0.01 | 0.02 | 0.34* |
| **BRI+BMI** | **0.736 (0.722 -0.750)** |  |  |  |  |  |  |  |  |  | NA | 0.002 | 0.01 | 0.34* |
| **Cindex+BMI** | **0.736 (0.722 -0.750)** |  |  |  |  |  |  |  |  |  |  | NA | 0.01 | 0.34* |
| **ABSI+BMI** | **0.736 (0.722-0.750)** |  |  |  |  |  |  |  |  |  |  |  | NA | 0.34* |
| **WC+Cindex** | 0.739 (0.725-0.759) |  |  |  |  |  |  |  |  |  |  |  |  | NA |

WC, Waist circumference; WHtR, Waist-to-Height Ratio; BMI, Body Mass Index; PI, Ponderal Index. C Index, Conicity Index; ABSI, A body Shape Index; BRI, Body Roundness Index; NA, not available; *, p< 0.05
