# Supplementary material for: Association between measures of adiposity and blood pressure levels in adult Cameroonians
Source: Health Sci Rep. 2021 May 3;4(2):e259. doi: 10.1002/hsr2.259 (PMC8093855; doi:10.1002/hsr2.259)
Supplement: Supplementary file 2 — Table S2 Odds ratios for a SD change measures and across quintiles of adiposity measures for predicting hypertension using average blood pressure adjusted for age and gender [file HSR2-4-e259-s004.docx]

| **Supplementary Table 2: Odds ratios for a standard deviation change measures and across quintiles of adiposity measures for predicting hypertension using average blood pressure adjusted for age and gender** | | | | | | | |
| --- | --- | --- | --- | --- | --- | --- | --- |
|  |  |  |  |  |  |  |  |
| **Variable** |  | **Q1** | **Q2** | **Q3** | **Q4** | **Q5** | **aOR (95%CI) of a SD change in AI** |
| **WC** | **Median (cm)** | 74 | 82 | 89 | 96 | 107 |  |
|  | **OR (95% CI)** | Ref | 1.274  (0.981 -1.660) | 1.561  (1.214 -2.015) | 2.180  (1.716 -2.786) | 2.906  (2.302 -3.692) | 1.440  (1.345 -1.543) |
| **WHtR** | **Median** | 0.4402 | 0.4904 | 0.5330 | 0.5769 | 0.6469 |  |
|  | **OR (95% CI)** | Ref | 1.343  (1.019 -1.778) | 2.205  (1.712 -2.858) | 2.175  (1.687 -2.821) | 3.403  (2.656 -4.390) | 1.399  (1.306 -1.499) |
| **BMI** | **Median (kg/m2)** | 20.97 | 23.92 | 26.53 | 29.31 | 34.48 |  |
|  | **OR (95% CI)** | Ref | 1.224  (0.994 -1.509) | 1.809  (1.492 -2.200) | 2.229  (1.843 -2.703) | 2.758  (2.292 -3.331) | 1.216  (1.163 -1.270) |
| **PI** | **Median (kg/m3)** | 12.31 | 14.22 | 15.86 | 17.70 | 21.28 |  |
|  | **OR (95% CI)** | Ref | 1.144  (0.933 -1.404) | 1.763  (1.457 -2.137) | 2.160  (1.795 -2.606) | 2.638  (2.189 - 3.187) | 1.107  (1.0578 -1.155) |
| **C index** | **Median (m2/3/kg1/2)** | 1.074 | 1.158 | 1.215 | 1.271 | 1.366 |  |
|  | **OR (95% CI)** | Ref | 1.172  (0.898 -1.534) | 1.555  (1.197 -2.029) | 1.809  (1.412 -2.330) | 2.060  (1.610 -2.651) | 1.215  (1.129 -1.308) |
| ***ABSI** | **Median (m7/6/kg2/3)** | 0.0845 | 0.0910 | 0.0951 | 0.0993 | 0.1064 |  |
|  | **OR (95% CI)** | Ref | 1.047  (0.806 -1.361) | 1.383  (1.085 -1.770) | 1.621  (1.277 -2.064) | 1.665  (1.321 -2.108) | 1.142  (1.062 -1.228) |
| **BRI** | **Median** | 2.299 | 3.155 | 3.997 | 4.911 | 6.537 |  |
|  | **OR (95% CI)** | Ref | 1.347  (1.021 -1.784) | 2.201  (1.703 -2.864) | 2.235  (1.732 -2.902) | 3.388  (2.637 -4.386) | 1.353  (1.269-1.444) |
| ****, P< 0.05 ; P> 0.05 for the rest. Ref, Reference; WC, Waist circumference; WHtR, Waist-to-Height Ratio; BMI, Body Mass Index; PI, Ponderal Index; C Index, Conicity Index; ABSI, A body Shape Index; BRI, Body Roundness Index, OR, Odds Ratio; aOR, Odds Ratio Adjusted for Age, gender. Q1, first quintile ; Q2, second quintile ; Q3, third quintile; Q4, fourth ; Q5, fifth quintile ; AI, Anthropometric index .*** | | | | | | | |
